# Modified Use of Classical Rein Technique in Laparoscopic Hysterectomy for Uterine Manipulation

**DOI:** 10.3390/medicina61122220

**Published:** 2025-12-16

**Authors:** Mert Cenker Güney, Selin Güney, Fatma Ceren Güner, Abdullah Boztosun

**Affiliations:** 1 Department of Obstetrics and Gynecology, Van Education & Research Hospital, Van 65300, Turkey; dr.mertcenkerguney.akd@gmail.com (M.C.G.);; 2 Clinic of Gynecologic Oncology, Isparta City Hospital, Ministry of Health, Isparta 32200, Turkey; 3 Department of Gynecology and Obstetrics, Akdeniz University, Antalya 07070, Turkey; abdullahboztosunyrd@hotmail.com

**Keywords:** laparoscopic hysterectomy, uterine manipulation, rein technique, Boztosun method, intra-abdominal tape, colpotomy time

## Abstract

*Background and Objectives*: Vaginal uterine manipulators facilitate laparoscopic hysterectomy but are limited by cost and anatomical constraints. The Boztosun method offers a cost-effective intra-abdominal alternative. This study evaluated the clinical performance and safety of this technique. *Materials and Methods*: This single-center, retrospective descriptive study analyzed 40 patients who underwent laparoscopic hysterectomy using the Boztosun method at Akdeniz University Hospital between October 2021 and June 2022. Clinical characteristics and perioperative outcomes were assessed. *Results*: The mean operative time was 78.5 ± 20.6 min, and the mean colpotomy time was 8.05 ± 3.57 min. Conversion to laparotomy occurred in 3 patients (7.5%), primarily due to extensive adhesions or large uterine size. No intraoperative complications, organ injuries, or blood transfusions were recorded. All patients were discharged within two days. Patients with prior abdominal surgery had significantly longer operative and colpotomy times (*p* < 0.05). *Conclusions*: The Boztosun method is a safe, efficient, and low-cost alternative to vaginal manipulators in laparoscopic hysterectomy. It may be particularly useful in resource-limited settings or when vaginal manipulation is not feasible.

## 1. Introduction

Hysterectomy is one of the most frequently performed operations by surgeons in gynecology [1]. The most common benign indications include abnormal uterine bleeding, chronic pelvic pain, pelvic organ prolapse, and symptomatic leiomyomas, with the primary goal of improving quality of life and preventing sexual dysfunction [2]. The impact of hysterectomy on a woman’s quality of life and sexual function can vary depending on factors such as preoperative mental health, baseline sexual function, surgical indication, and the technique used [3]. The indications for hysterectomy, however, are not limited to benign diseases and also encompass malignant and complex obstetric conditions. These include hysterectomy for endometrial cancer and urgent procedures such as cesarean hysterectomy, which may be required for morbidly adherent placenta or intractable postpartum hemorrhage, further broadening the scope of its clinical necessity [4,5,6].

Surgeons can perform hysterectomy using various techniques such as laparoscopic, abdominal, or vaginal routes. Among these, abdominal hysterectomy provides superior visualization and manipulation of pelvic organs. It is often preferred in cases involving large pelvic masses or extensive adhesions. However, abdominal hysterectomy is associated with higher rates of complications, such as hemorrhage, bladder injury, infection, and ureteral or rectal damage [7]. These risks are reported to be lower with laparoscopic hysterectomy, which also offers better cosmetic outcomes and faster postoperative recovery [7].

Uterine manipulators used during laparoscopic hysterectomy allow the surgeon to control uterine positioning, thereby facilitating a safer and technically more efficient procedure. Improved visualization and access achieved through manipulation also help reduce the risk of injury to adjacent structures such as the bladder, ureters, and rectum [8]. Elevating the uterus anteriorly and superiorly enables clearer exposure of the uterocervical junction. This allows for more precise colpotomy and preservation of vaginal tissue, which may contribute to better maintenance of sexual function after surgery [9].

Nevertheless, an ideal uterine manipulator has yet to be developed in terms of functional design. While certain models require specialized training, the high cost of many devices continues to limit their accessibility and routine use, particularly in settings with limited resources [10]. Considering the financial burden, accessibility issues, and technical challenges associated with vaginal uterine manipulators, intra-abdominal alternatives may offer practical advantages in selected cases. In this context, the classical Rein technique, previously introduced in our clinic, represents one such approach for intra-abdominal uterine manipulation [11].

In this study, we aimed to evaluate the effectiveness of an intra-abdominal uterine manipulation method, which is referred to as the Boztosun technique, representing a modification of the classical Rein method. The Boztosun technique was introduced to address specific technical challenges associated with the original Rein method, including the complexity of tying knots within the abdominal cavity, the risk of tape displacement during surgery, and its potential to stick to adjacent tissues due to intraoperative bleeding [11].

## 2. Materials and Methods

This retrospective descriptive study was conducted at the Department of Obstetrics and Gynecology, Akdeniz University School of Medicine, and included patients who underwent laparoscopic hysterectomy using the modified Rein technique between October 2021 and June 2022. The study was approved by the institutional ethics committee (Approval No: KAEK-459) and conducted in accordance with the principles of the Declaration of Helsinki.

In all patients included in the study, uterine manipulation was achieved without the use of a vaginal manipulator. Instead, a modified version of the classical Rein technique, previously introduced to the literature by our clinic, was employed—referred to herein as the Boztosun method.

**Figure 1:**
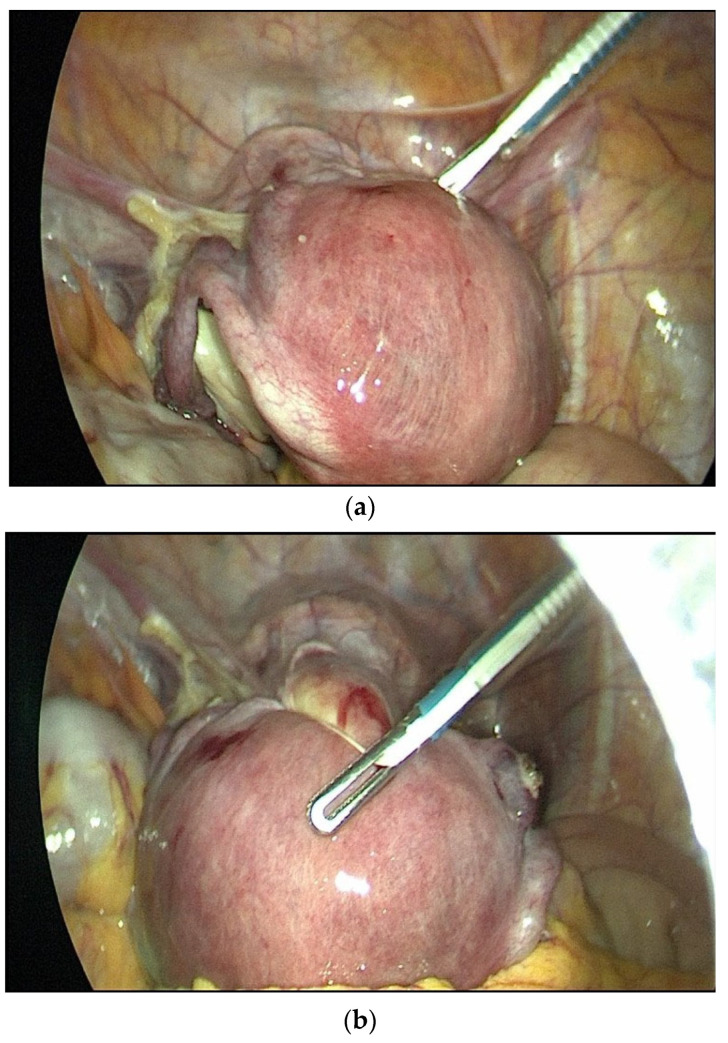

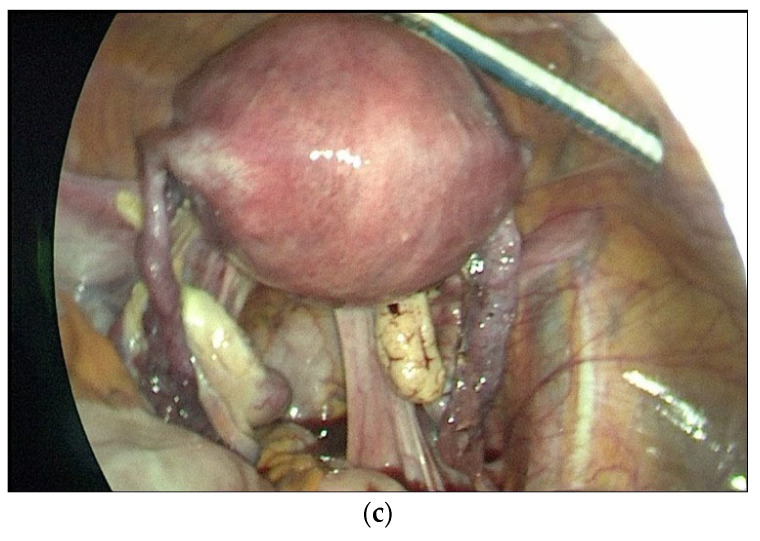
Lateral manipulation (**a**), anterior manipulation (**b**) and posterior manipulation (**c**) in the modified Rein technique (Boztosun Method).

The modified Rein method used in this study was adapted from a previously reported version by the same team [11,12]. In this modified Rein technique (Boztosun method), two polyester cotton tapes were looped and tied around the tips of grasper instruments (Figure 1). These were introduced into the abdominal cavity through 5 mm trocars. A second grasper was used to pass the looped tape around the uterus. The tapes were then tightened and secured externally, allowing uterine manipulation through movements of the grasper alone. During colpotomy, only the cup component of the Clermont-Ferrand uterine manipulator (Karl Storz^®^, Tuttlingen, Germany) was used as a guiding tool to identify the vaginal cuff incision site.

**Figure 2:**
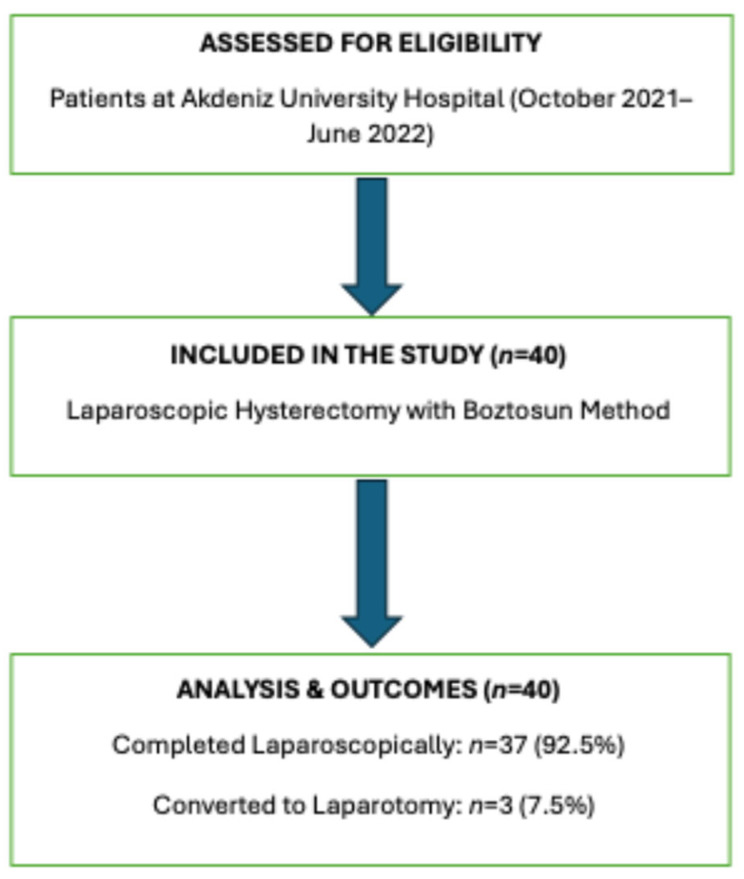
Study flowchart illustrating the patient selection process.

Patient data were obtained from electronic medical records and operative notes. The recorded variables included age, parity, body mass index (BMI), operative time, colpotomy time, uterine weight, preoperative and postoperative hemoglobin levels, drain placement, additional oophorectomy, intraoperative complications, need for conversion to laparotomy, blood transfusion requirement, and pathology results. A flowchart detailing the patient selection process is presented in Figure 2.

Descriptive statistics were presented as frequencies (*n*), percentages (%), means, standard deviations (SD), medians, and ranges. The normality of continuous variables was assessed using the Shapiro–Wilk test. Categorical variables were compared using Pearson’s chi-square or Fisher’s exact test. For continuous variables, the independent samples *t*-test was used when data were normally distributed, and the Mann–Whitney U test was applied otherwise. Correlations between continuous variables, such as uterine weight and BMI, and colpotomy time were evaluated using Pearson or Spearman correlation analysis as appropriate. The strength of the correlation was interpreted based on the r value as follows: 0.00–0.19 ‘very weak’, 0.20–0.39 ‘weak’, 0.40–0.59 ‘moderate’, 0.60–0.79 ‘strong’, and 0.80–1.0 ‘very strong’ [13].

All statistical analyses were performed using IBM SPSS Statistics version 23.0 (IBM Corp., Armonk, NY, USA). A *p*-value < 0.05 was considered statistically significant.

A post hoc power analysis was conducted using G*Power software (version 3.1.9.4) to determine the achieved statistical power of the study. Based on a point biserial correlation model with a total sample size of 40, a type I error rate of 0.05, and a medium-to-large effect size of 0.5, the analysis indicated an achieved power of 0.973 for a one-tailed test [14].

## 3. Results

This study evaluated 40 patients aged between 40 and 78 years, with a mean age of 50.17 ± 8.6 years. The average patient height, weight, and BMI were 159.93 ± 5.47 cm, 75.3 ± 12.38 kg, and 29.47 ± 5.28 kg/m^2^, respectively. The median uterine weight was 124.65 g (range: 38–469 g). Among the patients, 24 (60%) were postmenopausal, and 17 (42.5%) had a history of previous abdominal surgery. Detailed preoperative characteristics are presented in Table 1.

The mean operative time was 78.5 ± 20.55 min, and the mean colpotomy time was 8.05 ± 3.57 min. The median hemoglobin drop on postoperative day 1 compared to the preoperative period was 1.9 g/dL (range: −0.1 to 3.8 g/dL). Drains were placed in 8 patients (20%). In 21 cases (52.5%), oophorectomy was performed during the procedure.

Conversion to laparotomy was required in 3 out of 40 patients (7.5%). In two cases, dense intra-abdominal adhesions due to prior surgeries prevented safe laparoscopic dissection, while in one case, laparotomy was preferred due to a large uterus making specimen removal difficult via the laparoscopic route. The majority of surgeries were performed for benign gynecologic indications, most commonly abnormal uterine bleeding and myoma uteri. No patient experienced intraoperative bladder, ureteral, bowel, or vascular injuries, and no transfusions were needed at any stage. All bladder catheters were removed on the first postoperative day, and all patients received prophylactic antibiotics. No postoperative complications such as wound infections or urinary tract infections were observed. These findings are summarized in Table 2.

**Figure 3:**
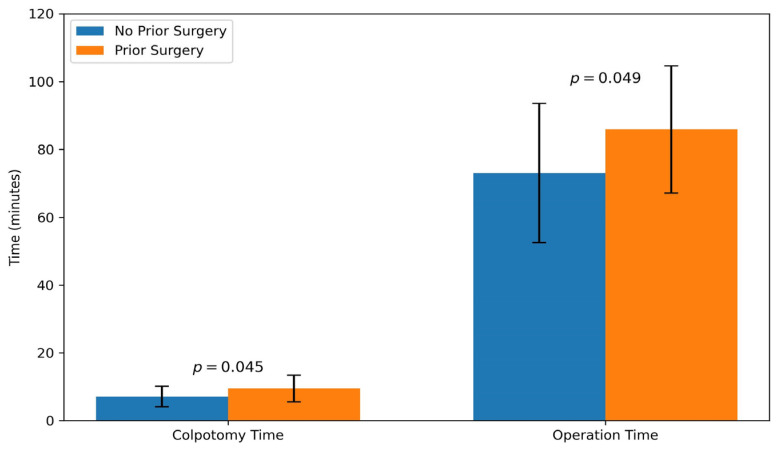
Comparison of colpotomy time and total operative time between patients with and without a history of abdominal surgery. Bars represent mean values ± standard deviation.

Colpotomy and total operation times were compared between patients with and without a history of abdominal surgery. The mean colpotomy time was significantly longer in those with previous abdominal surgery (9.47 ± 3.91 vs. 7.09 ± 3.04 min; *p* = 0.045). Similarly, the mean total operative time was also longer in this group (85.88 ± 18.71 vs. 73.04 ± 20.51 min; *p* = 0.049) (Figure 3).

Mean colpotomy time was 7.09 ± 3.04 min in patients without prior surgery (*n* = 23) and 9.47 ± 3.91 min in those with prior surgery (*n* = 17) (*p* = 0.045). Mean operation time was 73.04 ± 20.51 min in patients without prior surgery and 85.88 ± 18.71 min in those with prior surgery (*p* = 0.049).

**Figure 4:**
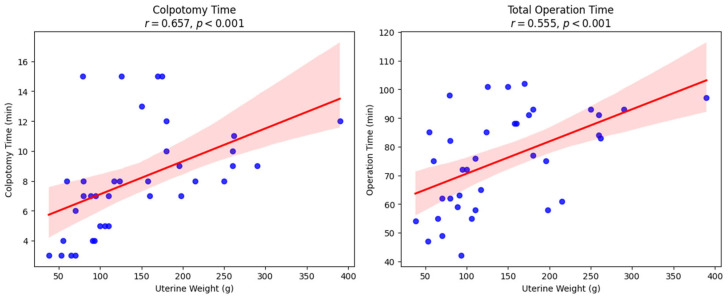
Correlation of uterine weight with colpotomy time and total operative time. Scatter plots showing the correlation between uterine weight and operative parameters.

Correlation analysis revealed a strong positive correlation between uterine weight and colpotomy time (r = 0.657; *p* < 0.001) and a significant positive correlation between uterine weight and total operation time (r = 0.555; *p* < 0.001). No significant correlations were observed between BMI and either surgical time parameter (Figure 4).

The left panel presents the relationship between uterine weight and colpotomy time (r = 0.657, *p* < 0.001), and the right panel displays the correlation between uterine weight and total operation time (r = 0.555, *p* < 0.001). Both associations were statistically significant. Each dot represents an individual patient, and the red line indicates the fitted linear regression trend and the shaded red area represents the 95% confidence interval for the regression line.

The relationship between laparotomy conversion and patients’ uterine weight and history of previous abdominal surgery is presented in Table 3. Although the median uterine weight was higher in patients requiring laparotomy (169 g vs. 117 g), the difference was not statistically significant (*p* = 0.192). Similarly, previous abdominal surgery was more common in the laparotomy group (66.7% vs. 40.5%), but this difference was not significant (*p* = 0.565).

## 4. Discussion

Although the clinical indications for hysterectomy are well-established, the surgical approach has significantly evolved from open surgery towards minimally invasive techniques due to their proven benefits in postoperative recovery and reduced morbidity [2,7,15]. However, the widespread adoption of laparoscopic hysterectomy is often hindered by the technical complexity and high costs associated with commercial vaginal uterine manipulators [10,16,17]. Consequently, there is a growing necessity for cost-effective, manipulator-free intra-abdominal techniques that maintain surgical safety and efficiency, particularly in resource-limited settings [11,12].

As summarized in Table 4, Given the high cost and accessibility issues of standard vaginal manipulators, manipulator-free intra-abdominal solutions, such as the Classic Rein Technique, Uterine Hitch, and Kavallaris techniques, are crucial alternatives, especially in resource-limited settings. While these methods are cost-effective, many present challenges, such as difficulties with intra-abdominal knot-tying or the risk of tape displacement reported with the Classic Rein technique. The Boztosun Technique, a modification of the Classic Rein method, provides high stability and control at a very low cost by utilizing a looped cotton tape secured by a grasper. This modification addresses technical drawbacks, making placement and repositioning simpler and contributing to the shorter operative and colpotomy times observed in our study [10,11,12].

In this study, we evaluated laparoscopic hysterectomy cases performed using the Boztosun technique, a modified version of the Rein method that does not require the use of a vaginal uterine manipulator. The mean operative time was found to be 78.5 ± 20.55 min. In the literature, similar procedures performed with Clermont-Ferrand and Vectec manipulators have reported operative times of 89 and 81 min, respectively [17]. Another study from Italy reported a mean operative time of 84 min for laparoscopic hysterectomy [16]. These findings suggest that the Boztosun technique yields comparable operative times to those reported in the literature.

Colpotomy duration is a key indicator for assessing both surgical efficiency and the effectiveness of the uterine manipulator used. In a multicenter study conducted in Turkey in 2019, the mean colpotomy time was reported as 10.3 min with the Mangeshikar manipulator and 8.8 min with the V-Care manipulator [18]. Another study comparing the Clermont Ferrand and Vectec manipulators found colpotomy durations of 4.5 and 4 min, respectively [17]. In the Classical Rein Technique previously applied in our clinic, the mean colpotomy duration was 10.2 min, whereas in the present study using the Modified Rein Technique, this duration was reduced to 8.05 ± 3.57 min [11]. Although the classical technique data were derived from a previously published independent study, a post hoc analysis comparing the two methods yielded a Cohen’s d effect size of 0.775 and a statistical power of 0.868. These findings suggest that the modification introduced in the technique provided a statistically significant and clinically meaningful improvement in reducing colpotomy time.

Postoperative hemoglobin reduction is considered an indirect indicator of intraoperative blood loss. In our study, the mean decrease in hemoglobin was calculated as 1.01 units. When compared to the previously reported mean reduction of 1.4 units associated with the Classical Rein Technique, this lower value suggests that the Modified Rein Technique may offer an advantage in terms of minimizing blood loss [11].

Length of hospital stay is an important indicator of both surgical efficiency and the patient’s postoperative recovery. In a large-scale study conducted in the United States, the rate of hospital stays longer than three days following laparoscopic hysterectomy was reported as 2.8 percent, while this rate increased to 42 percent among patients who underwent abdominal hysterectomy. None of the patients required hospitalization beyond two days. The difference was found to be statistically significant [19]. Another study also reported that the length of stay after laparoscopic hysterectomy most often ranged between one and two days [20]. In our study, 47.5 percent of patients who underwent surgery using the Boztosun technique were discharged on the first postoperative day, and 52.5 percent were discharged on the second day. None of the patients required hospitalization beyond two days.

In certain cases, laparoscopic hysterectomy may not be completed as planned and conversion to open surgery becomes necessary. This may occur due to intraoperative complications or when the uterus or adnexal mass is too large to be safely removed using laparoscopic instruments. In the literature, reported conversion rates from laparoscopy to laparotomy range from 1.7% to 10.9% [20,21]. In our study, the conversion rate was 7.5%. Among the three patients who required laparotomy, one case involved a uterus too large to be extracted laparoscopically, while in the remaining two cases, dense pelvic adhesions from previous surgeries were the primary reason for conversion. In a previous series conducted in our clinic using the Classical Rein Technique, the success rate was reported as 93.1%. In comparison, the Boztosun technique achieved a success rate of 92.5% in the present study, indicating comparable outcomes between the two methods [11].

In terms of complications, a large series involving 3090 patients reported intraoperative and postoperative complication rates of 1.4% and 13.9%, respectively [20]. In our series using the Boztosun technique, no complications were observed. While this finding supports the safety of the method, the relatively small sample size limits the generalizability of this result.

In cases where a vaginal uterine manipulator is unavailable or no team member is proficient in its use, this approach offers a significant advantage in terms of the sustainability of minimally invasive hysterectomy. The ability to manipulate the uterus intra-abdominally may enable the surgeon to perform the procedure safely and effectively, even with a less experienced surgical team. Additionally, the ease and speed with which the Cotton Tape can be applied around the uterus, as well as its potential for reapplication when needed, may contribute to improved intraoperative efficiency. Compared to the classical Rein technique, this method is notable for its shorter total operative and colpotomy times, lower postoperative hemoglobin decline, and comparable overall success rate.

Our study possesses several key strengths. The Boztosun technique offers a safe, efficient, and highly cost-effective alternative to expensive standard manipulators. Furthermore, our post hoc analysis demonstrated a statistically and clinically significant reduction in colpotomy time compared to the classic rein technique, confirming the value of our modification. The rapid length of hospital stay (all patients discharged within two days) further supports the method’s high safety profile. However, the primary limitations include the relatively small sample size and the retrospective, single-center design, which may limit generalizability. Future prospective, multicenter studies are necessary to confirm the technique’s reproducibility among less experienced surgeons.

## 5. Conclusions

The modified rein technique (Boztosun method), which we developed inspired by the classic rein technique, demonstrated great benefits in the manipulation of the uterus and during colpotomies. The most challenging situation for utilizing the classic rein technique was when the cotton tape is attached to and exits the uterus. Thanks to the method we have developed, the cotton tape is attached to the uterus much faster and easier, and it is simpler to reattach in case of possible dislocation. This advantage facilitated the applicability of the method. When the operation time, colpotomy time, postoperative hemogram reduction, discharge time, complications, and the success of the method were compared, the results were found to be significant compared to the classic rein technique, as well as the results reported in much of the relevant literature.

## Figures and Tables

**Table 1:** Preoperative Characteristics of the Patients.

Variable	Mean ± SD	Median (Min–Max)
Age (years)	50.17 ± 8.6	49 (40–78)
Height (cm)	159.93 ± 5.47	158.5 (153–179)
Weight (kg)	75.3 ± 12.38	75.5 (47–103)
BMI (kg/m^2^)	29.47 ± 5.28	29.25 (19–42.8)
Uterine weight (g)	151.77 ± 92.73	124.65 (38–469)
	***n***	**%**
Menopausal status		
Premenopausal	16	40.0
Postmenopausal	24	60.0
Prior abdominal surgery		
Absent	23	57.5
Present	17	42.5

**Table 2:** Intraoperative and Postoperative Characteristics.

Variable	Mean ± SD	Median (Min–Max)
Operation duration (min)	78.5 ± 20.55	79.5 (42–132)
Colpotomy duration (min)	8.05 ± 3.57	8 (3–15)
Hemoglobin drop—Day 1 (g/dL)	1.74 ± 1	1.9 (−0.1–3.8)
	***n***	**%**
Drain placed	8	20.0
Completed laparoscopically		
No (Laparotomy)	3	7.5
Yes	37	92.5
Prophylactic antibiotics	40	100.0
Postoperative complications	0	0
Duration of hospital stay		
1 day	19	47.5
2 days	21	52.5

**Table 3:** Uterine Weight and Abdominal Surgery History in Patients Who Underwent Laparotomy.

Variable	Laparotomy (*n* = 3)	No Laparotomy (*n* = 37)	*p*-Value
Uterine weight (g), median (min–max)	169 (128–469)	117 (38–390)	0.192
Prior abdominal surgery *n* (%)			0.565
Absent	1 (33.3)	22 (59.5)	
Present	2 (66.7)	15 (40.5)	

**Table 4:** Cost–Benefit Comparison of Manipulator-Free Methods for Uterine Manipulation in Laparoscopic Hysterectomy.

Technique	Key Mechanism	Cost Implication	Control & Stability	Primary Limitation
Boztosun Technique	Cotton tape looped via a grasper (intra-abdominal)	Very Low	High	Initial learning curve for the modification
Classic Rein Technique	Suture/Tape tied around the uterine fundus (intra-abdominal)	Very Low	Medium–High	Technical difficulty of intra-abdominal knotting; risk of slippage/adherence
Uterine Hitch Technique	Suture/Tape secured through the vaginal fornix	Low	Medium	Limited range of motion and traction
Kavallaris Technique	Use of specialized forceps or tenaculum	Low/Medium	Medium	Requires continuous assistant involvement; risk of slippage

## Data Availability

The data presented in this study are available on request from the corresponding authors due to privacy and ethical considerations.

## Abbreviations

The following abbreviations are used in this manuscript:

BMI	Body Mass Index
SD	Standard Deviation
SPSS	Statistical Package for the Social Sciences
